# The polarization problem for the honeycomb structure

**DOI:** 10.1007/s00013-026-02300-8

**Published:** 2026-10-24

**Authors:** Markus Faulhuber

**Affiliations:** Faculty of Mathematics, University of Vienna, Oskar-Morgenstern-Platz 1, 1090 Vienna, Austria

**Keywords:** Hexagonal lattice, Honeycomb structure, Periodic configuration, Polarization, 52C25, 74G65

## Abstract

We study the polarization problem in dimension 2 for the honeycomb structure and compare it to the maximal polarization lattice, the hexagonal lattice. As expected, the hexagonal lattice has higher polarization than the honeycomb at all densities.

## Introduction and notation

The *polarization problem* has its origin in potential analysis, and the study of Chebyshev constants and the work of Fekete [17] and Polyá and Szegő [22]. It has been intensively studied in recent years for the Riesz potential on the (*d*-dimensional) sphere, see, e.g., [1, 9, 10, 12, 19], to name just a few articles. We consider the problem for periodic point configurations in the plane and for exponential potentials of squared distance, i.e., Gaussians. A problem for potentials $f(r^2)$ with $f:{\mathbb {R}}_+ \rightarrow {\mathbb {R}}_+$, being decreasing and convex, was formulated for lattices by Hardin, Petrache, and Saff [20] and answered by Bétermin, Faulhuber, and Steinerberger [8]. Most recently, Bachoc, Moustrou, Vallentin, and Zimmermann characterized *cold spots* of many root lattices [2], which is a first step towards tackling the polarization problem in ${\mathbb {R}}^d$.

We call $f: {\mathbb {R}}_+ \rightarrow {\mathbb {R}}_+$, r↦f(r), completely monotone if $(-1)^k f^{(k)}(r) \ge 0$ and of sufficiently fast decay if $f(r)=\mathcal {O} (r^{-\frac{d}{2}-\varepsilon })$, ε>0. We will consider the following problem:

1.1 $$\begin{aligned} \max _{\Gamma \subset {\mathbb {R}}^d} \min _{z \in {\mathbb {R}}^d} \sum _{\gamma \in \Gamma } f(|\gamma -z|^2). \end{aligned}$$

This is known as the polarization problem in Euclidean space ${\mathbb {R}}^d$. The minimum depends generally on the geometry and density (defined below) of Γ, and the potential *f*. The maximizer among (periodic) point configurations Γ is sought for a fixed density of Γ. In rare cases, a maximizer may be optimal among all densities (scales), in which case it becomes a universal polarization maximizer, much like universally optimal energy minimizers in the sense of Cohn and Kumar [13]. For energy minimization, solutions have been derived in dimensions 8 and 24 by Cohn, Kumar, Miller, Radchenko, and Viazovska [14]. The energy minimization problem in dimension 2 was solved among lattices by Montgomery [21]. For polarization, we only know that the hexagonal lattice $\Lambda _2$ (the $\textsf{A}_2$ root lattice) is a universal polarization maximizer among lattices (of any fixed density) and completely monotone potentials of squared distance [8]. In this note, we prove the following polarization result.

### Theorem 1.1

Denote the hexagonal lattice by $\Lambda _2$ and the honeycomb structure by $\Gamma _2$, both normalized to density 1. Let ρ>0 be fixed and let $f: {\mathbb {R}}_+ \rightarrow {\mathbb {R}}_+$ be completely monotone of squared distance and of sufficiently fast decay. The hexagonal lattice scaled to density ρ has higher polarization than the honeycomb structure scaled to density ρ:

$$\begin{aligned} \min _{z} \sum _{\lambda \in \rho ^{-1/2}\Lambda _2} f(|\lambda -z|^2) > \min _{z} \sum _{\lambda \in \rho ^{-1/2}\Gamma _2} f(|\gamma -z|^2). \end{aligned}$$

By the Bernstein–Widder theorem [5, 23], a completely monotone function of squared distance can be written as

1.2 $$\begin{aligned} f(r^2) = \mathop {\int \limits _{{\mathbb {R}}_+}} e^{-\alpha r^2} \, d \mu _f(\alpha ) \end{aligned}$$

for a non-negative Borel measure $\mu _f$. In accordance with [2], we say a structure Γ has a cold spot in $z_0$ if (fix *f* for the moment) $z_0$ is a minimizer in (1.1). It is a universal cold spot if it does not depend on the density of Γ. For the potential $\phi _\alpha (r^2) = e^{-\pi \alpha r^2}$, if Γ solves (1.1) for any fixed density (which corresponds to being a maximizer at density 1 and all scales α>0 of the Gaussian), then it is a universal polarization maximizer for all completely monotone functions of squared distance $f(r^2)$ (given fast enough decay of *f*). In this case, the universal cold spot is also independent of *f*. Yet, the existence of universally polarization maximizers is open, except for lattices in dimension 2 [8].

A (full-rank) lattice Λ in ${\mathbb {R}}^d$ is a discrete co-compact subgroup (see [15] for a full introduction) and its density is the average number of lattice points per volume, given by

$$\begin{aligned} \rho (\Lambda ) = 1/\textrm{vol}({\mathbb {R}}^d/\Lambda ). \end{aligned}$$

A periodic configuration is the union of relatively shifted copies of a lattice $\Lambda \subset {\mathbb {R}}^d$;

$$\begin{aligned} \Gamma = \bigcup _{n=1}^N (\Lambda + x_n), \quad x_m-x_n \notin \Lambda , m \ne n. \end{aligned}$$

The density of Γ is then given by $\rho (\Gamma ) = N/\textrm{vol}({\mathbb {R}}^d/\Lambda )$ (cf. [13, Sect. 9]).

## Universal cold spots and the honeycomb structure

**Fig. 1:**
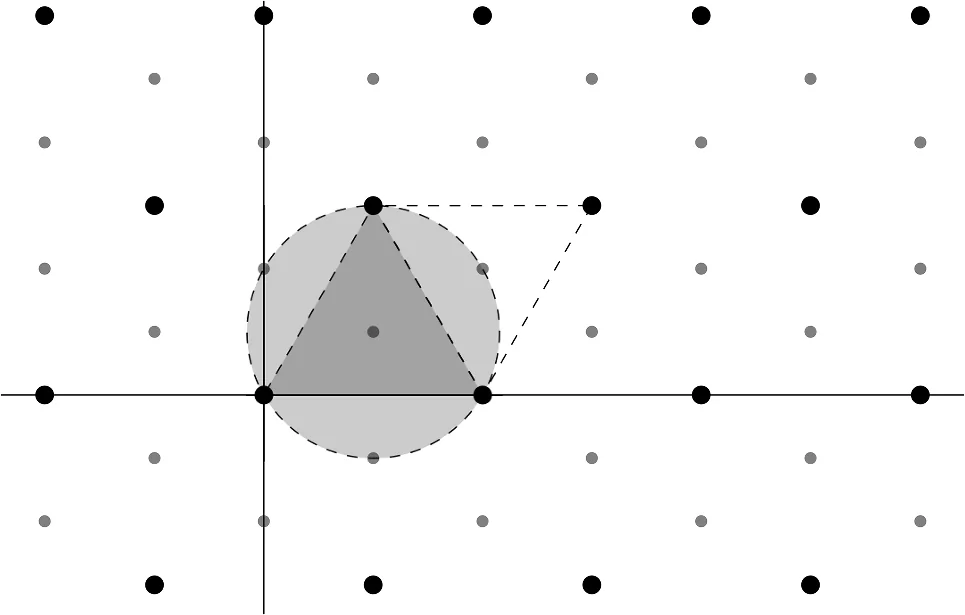
A part of the hexagonal lattice $\Lambda _2$ with its fundamental cell, a fundamental triangle, and a covering circle centered at a deep hole. The large black dots are the lattice points, and the smaller gray dots are deep holes

The honeycomb $\Gamma _2$ structure is the union of two hexagonal lattices, relatively shifted by a deep hole $z_0$. A deep hole of a lattice is a point in ${\mathbb {R}}^d$ maximizing the distance to the closest lattice point(s). For the hexagonal lattice, it is the circumcenter of the equilateral (fundamental) triangle formed by 3 lattice points (see Fig. 1).

The hexagonal lattice of density 1 is, up to rotational equivalence, explicitly given by

$$\begin{aligned} \Lambda _2 = \sqrt{\frac{1}{\sin (\pi /3)}} \begin{pmatrix} 1 &  \cos (\pi /3)\\ 0 &  \sin (\pi /3) \end{pmatrix} {\mathbb {Z}}^2 = \sqrt{\tfrac{2}{\sqrt{3}}} \begin{pmatrix} 1 &  \frac{1}{2}\\ 0 &  \frac{\sqrt{3}}{2} \end{pmatrix} {\mathbb {Z}}^2. \end{aligned}$$

The honeycomb structure of unity density is given by

2.1 $$\begin{aligned} \Gamma _2 = \sqrt{2} \left( \Lambda _2 \cup (\Lambda _2+z_0) \right) . \end{aligned}$$

The factor $\sqrt{2}$ scales $\Gamma _2$ appropriately and we always write $z_0$ for the deep hole(s) of $\Lambda _2$.

For the hexagonal lattice $\Lambda _2$ and the potential $\phi _\alpha (r) = e^{-\pi \alpha r}$ (the π in the exponent is for technical reasons, due to our normalization (3.2) of the Fourier transform), we have

2.2 $$\begin{aligned} \sum _{\lambda \in \Lambda _2} \phi _\alpha (|\lambda -z_0|^2) \le \sum _{\lambda \in \Lambda _2} \phi _\alpha (|\lambda -z|^2), \quad \forall \alpha > 0, \ z \in {\mathbb {R}}^2. \end{aligned}$$

This result is due to Baernstein [3] and shows that deep holes are universal cold spots of the hexagonal lattice. Note that if $z_0 \in {\mathbb {R}}^2/\Lambda _2$ is a deep hole, then $2 z_0 \notin \Lambda _2+z_0$ is also a deep hole. These two deep holes are inequivalent; all other deep holes can be reached from these by translation by $\Lambda _2$. For the honeycomb structure $\Gamma _2$, we have, by setting $\widetilde{z} = \sqrt{2} z$,

2.3 $$\begin{aligned} \sum _{\gamma \in \Gamma _2} \phi _\alpha (|\gamma - \widetilde{z}|^2)= &   \sum _{\lambda \in \Lambda _2} \phi _\alpha (2|\lambda -z|^2) + \sum _{\lambda \in \Lambda _2} \phi _\alpha (2|\lambda +z_0-z|^2) \nonumber \\= &   \sum _{\lambda \in \Lambda _2} \phi _{2\alpha }(|\lambda -z|^2) + \sum _{\lambda \in \Lambda _2} \phi _{2\alpha }(|\lambda +z_0-z|^2). \end{aligned}$$

Note that scaling the arguments is necessary, due to (2.1). The scaling is put into the parameter α of the Gaussian $\phi _\alpha (r^2)$. This will also happen in sequential computations.

**Fig. 2:**
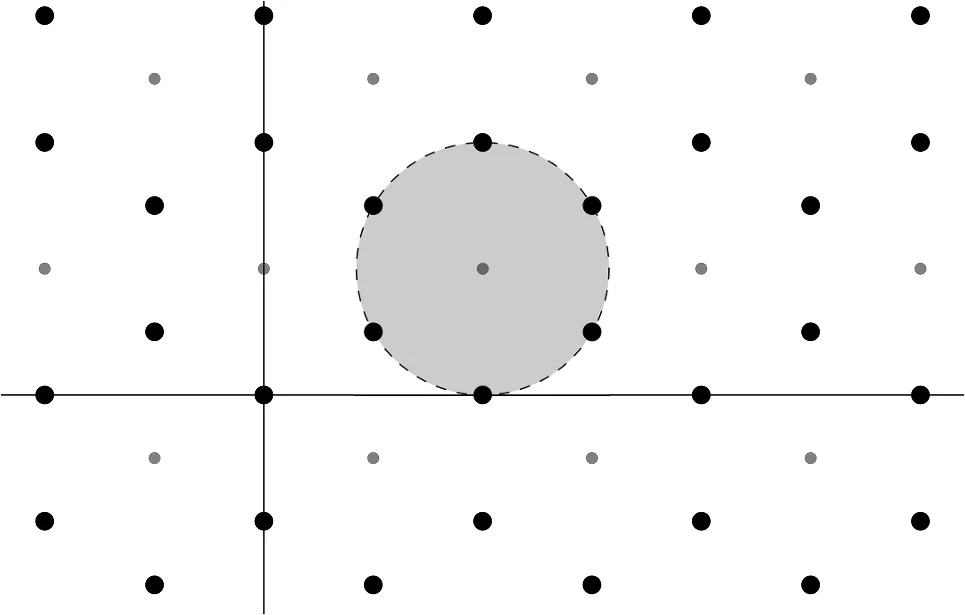
The honeycomb structure $\Gamma _2$ is a union of the hexagonal lattice and a copy shifted by a deep hole. The second, inequivalent deep hole of $\Lambda _2$ is a deep hole of $\Gamma _2$. The big black dots are points of $\Gamma _2$ while the smaller gray dots are its deep holes

As $2 z_0$ is a deep hole of $\Lambda _2$ and of $\Lambda _2+z_0$ (corresponding to $z_0$ of $\Lambda _2$), we have by (2.2)

$$\begin{aligned} \sum _{\gamma \in \Gamma _2} \phi _\alpha (|\gamma - 2 \widetilde{z}_0|^2) \le \sum _{\gamma \in \Gamma _2} \phi _\alpha (|\gamma - z|^2), \quad \forall \alpha > 0, \ z \in {\mathbb {R}}^2, \end{aligned}$$

where $\widetilde{z}_0 = \sqrt{2} z_0$ is a deep hole of the scaled hexagonal lattice $\sqrt{2} \Lambda _2$ (Fig. 2).

## Proof or Theorem 1.1

Most methods we use are basic, the Poisson summation formula being already an advanced technique. However, we build on deep insights by Baernstein [3] on the universality of cold spots in the hexagonal lattice. At one point, we will use a result of Ramanujan (see [4, Chap. 33]), put on solid mathematical ground by Borwein and Borwein [11].

By (1.2), it suffices to consider the potential $\phi _\alpha (|r^2|)$ and to show that

3.1 $$\begin{aligned} \sum _{\gamma \in \Gamma _2} \phi _{\alpha }(|\gamma - 2 \widetilde{z}_0|^2) < \sum _{\lambda \in \Lambda _2} \phi _\alpha (|\lambda -z_0|^2), \quad \forall \alpha > 0, \end{aligned}$$

in order to prove Theorem 1.1. We start with noting that

$$\begin{aligned} \sum _{\lambda \in \Lambda _2} \phi _\alpha (|\lambda -2z_0|^2) = \sum _{\lambda \in \Lambda _2} \phi _\alpha (|\lambda -z_0|^2) \end{aligned}$$

as both $z_0$ and $2z_0$ are deep holes of $\Lambda _2$ and, thus, universal cold spots [3]. We rewrite the entities from (3.1) over the integer lattice ${\mathbb {Z}}^2$ by a linear coordinate change (cf. [8, 21]):

$$\begin{aligned} \sum _{\lambda \in \Lambda _2} \phi _\alpha (|\lambda -z_0|^2)&= \sum _{(k,l) \in {\mathbb {Z}}^2} e^{- \frac{2 \pi \alpha }{\sqrt{3}} ((k-1/3)^2 + (k-1/3)(l-1/3)+(l-1/3)^2)}, \\ \sum _{\gamma \in \Gamma _2} \phi _{\alpha }(|\gamma - 2 \widetilde{z}_0|^2)&= \sum _{\lambda \in \Lambda _2} \phi _{2\alpha }(|\lambda -2 z_0|^2) + \sum _{\lambda \in \Lambda _2} \phi _{2\alpha }(|\lambda -z_0|^2) \\&= 2 \sum _{\lambda \in \Lambda _2} \phi _{2\alpha }(|\lambda -z_0|^2)\\&= 2 \sum _{(k,l) \in {\mathbb {Z}}^2} e^{- \frac{4 \pi \alpha }{\sqrt{3}} ((k-1/3)^2 + (k-1/3)(l-1/3)+(l-1/3)^2)}. \end{aligned}$$

To split the series for the honeycomb, we used (2.3). It is not hard to observe that

$$\begin{aligned} \min _{(k,l) \in {\mathbb {Z}}^2} \left( (k-1/3)^2 + (k-1/3)(l-1/3)+(l-1/3)^2\right) = 1/3, \end{aligned}$$

which is achieved if and only if

$$\begin{aligned} (k,l) \in \{(0,0),(1,0),(0,1)\}. \end{aligned}$$

As a next step, we note that for $x \in {\mathbb {R}}_+$,

$$\begin{aligned} e^{-x}> 2 e^{-2x} \quad \Longleftrightarrow \quad x > \log (2). \end{aligned}$$

This particularly implies that for all $(k,l) \in {\mathbb {Z}}^2$,

$$\begin{aligned}&e^{- \frac{2 \pi \alpha }{\sqrt{3}} ((k-1/3)^2 + (k-1/3)(l-1/3)+(l-1/3)^2)} \\&\quad> 2 e^{- \frac{4 \pi \alpha }{\sqrt{3}} ((k-1/3)^2 + (k-1/3)(l-1/3)+(l-1/3)^2)}\\ \quad \Longleftrightarrow \quad&\frac{2 \pi \alpha }{3 \sqrt{3}}> \log (2)\\ \quad \Longleftrightarrow \quad&\alpha > \frac{3 \sqrt{3} \log (2)}{2 \pi } \approx 0.573228. \end{aligned}$$

It readily follows that (3.1) holds for α large enough:

$$\begin{aligned} \sum _{\lambda \in \Lambda _2} \phi _\alpha (|\lambda -z_0|^2)> \sum _{\gamma \in \Gamma _2} \phi _\alpha (|\gamma -2 \widetilde{z}_0|^2), \quad \forall \alpha > \frac{3 \sqrt{3} \log (2)}{2 \pi }. \end{aligned}$$

To show that the polarization of $\Lambda _2$ is larger than that of $\Gamma _2$ on small scales α (equivalently for high densities), we employ the Poisson summation formula as usual in such situations.

We recall that the dual lattice $\Lambda ^*$ is characterized by $\Lambda ^* = \{ \lambda ^* \in {\mathbb {R}}^d \mid \lambda \cdot \lambda ^* \in {\mathbb {Z}}\ \forall \lambda \in \Lambda \}$, the dot · denoting the Euclidean inner product. The Fourier transform of $F: {\mathbb {R}}^d \rightarrow \mathbb {C}$ is

3.2 $$\begin{aligned} \widehat{F}(y) = \mathop {\int \limits _{{\mathbb {R}}^d}} F(x) e^{-2 \pi i x \cdot y} \, dx. \end{aligned}$$

The Poisson summation formula, in this case, reads

$$\begin{aligned} \sum _{\lambda \in \Lambda } F(\lambda +z) = \frac{1}{\textrm{vol}({\mathbb {R}}^d/\Lambda )} \sum _{\lambda ^* \in \Lambda ^*} \widehat{F}(\lambda ^*) e^{2 \pi i \lambda ^* \cdot z}, \quad z \in {\mathbb {R}}^d. \end{aligned}$$

Our situation is special; any lattice $\Lambda \subset {\mathbb {R}}^2$ (of density 1) is self-dual. More precisely, if $\Lambda \subset {\mathbb {R}}^2$, $\textrm{vol}({\mathbb {R}}^2/\Lambda ) = 1$, then $\Lambda ^* = J \Lambda$ (cf. [8, App. A]), where

$$\begin{aligned} J = \begin{pmatrix} 0 &  1\\ -1 &  0 \end{pmatrix} \end{aligned}$$

is a rotation by 90 degrees. Generally, the Gaussian $x \mapsto e^{-\pi |x|^2}$, $x \in {\mathbb {R}}^d$, is an eigenfunction of the Fourier transform. In particular, we have [18, App. A]

$$\begin{aligned} \widehat{\phi _\alpha }(y^2) = \alpha ^{-d/2} \phi _{1/\alpha }(y^2), \quad y \in {\mathbb {R}}^d. \end{aligned}$$

So, in our very particular situation, we have (note that $-\lambda \in \Lambda$ for any lattice)

$$\begin{aligned} \sum _{\lambda \in \Lambda _2} \phi _{\alpha }(|\lambda -z_0|^2) = \sum _{\lambda \in \Lambda _2} \phi _{\alpha }(|\lambda +z_0|^2) = \alpha ^{-1} \sum _{\lambda \in \Lambda _2} \phi _{1/\alpha }(|\lambda |^2) e^{2 \pi i (J\lambda ) \cdot z_0}. \end{aligned}$$

**Fig. 3:**
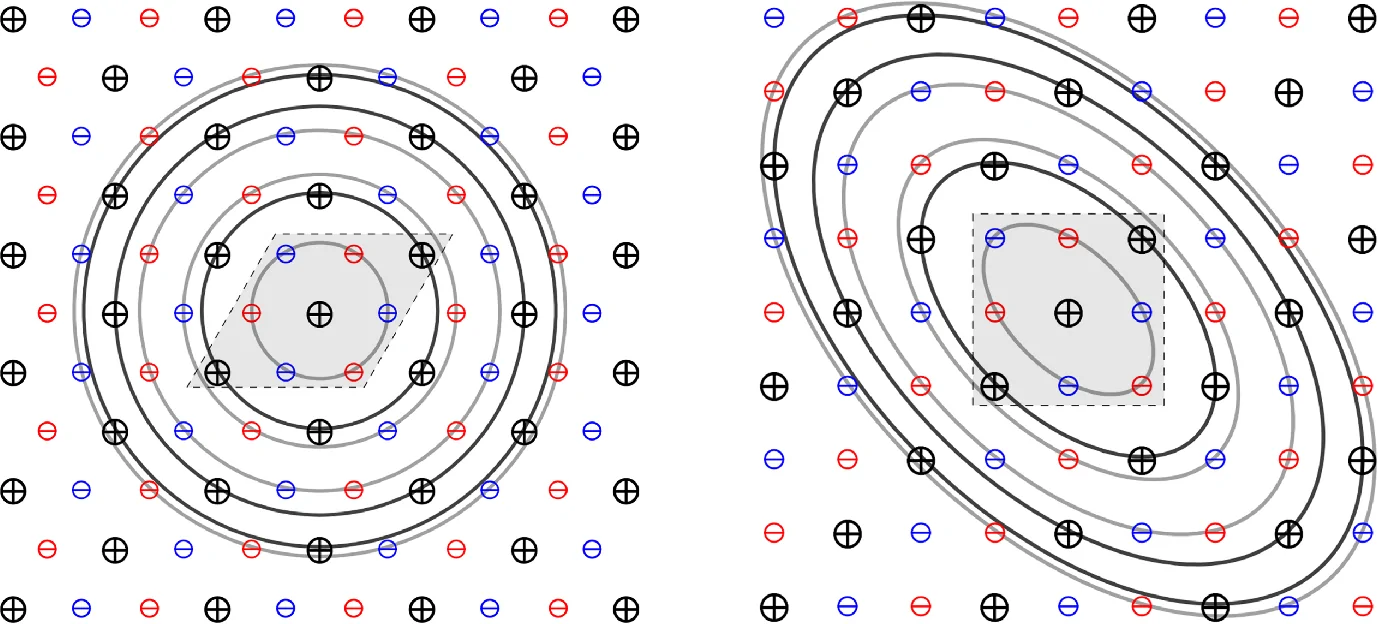
Left: After performing a Poisson summation, the polarization problem becomes a question of charged ionic crystals. The hexagonal lattice can then be decomposed into 3 (shifted) hexagonal lattices, each of third the density, 2 having negative charges of half weights and one having positive charges. Right: After a linear change of coordinates, the hexagonal setup is transferred to an anisotropic interaction problem on ${\mathbb {Z}}^2$. The anisotropic interaction is determined via the quadratic form $q(k,l)=k^2+kl+l^2$. In the square $[-1,1]^2$, we find twice as many negative charges of half weights as positive ones

We used the symmetry of $\Lambda _2$, the Poisson summation formula, the fact that $\textrm{vol}({\mathbb {R}}^2/\Lambda _2)=1$, that $\Lambda _2^* = J \Lambda _2$, and that $\phi _\alpha (|r|^2)$ is rotational invariant. Note that the left side is real-valued, so the right side must also be real-valued. With the same arguments, we obtain a dual formula for the polarization of $\Gamma _2$ (Fig. 3):

$$\begin{aligned} \sum _{\gamma \in \Gamma _2} \phi _{\alpha }(|\gamma - 2 \widetilde{z}_0|^2) = 2 \sum _{\lambda \in \Lambda _2} \phi _{2\alpha }(|\lambda +z_0|^2) = \alpha ^{-1} \sum _{\lambda \in \Lambda _2} \phi _{1/(2\alpha )}(|\lambda |^2) e^{2 \pi i (J\lambda ) \cdot z_0}. \end{aligned}$$

Thus, by making the substitution $\alpha \mapsto \alpha ^{-1}$, we seek to show that

$$\begin{aligned}  &   \sum _{\lambda \in \Lambda _2} \phi _{\alpha }(|\lambda |^2) e^{2 \pi i (J\lambda ) \cdot z_0}> \sum _{\lambda \in \Lambda _2} \phi _{\alpha /2}(|\lambda |^2) e^{2 \pi i (J\lambda ) \cdot z_0}, \quad \\    &   \alpha > \frac{2 \pi }{3 \sqrt{3} \log (2)} \approx 1.74451. \end{aligned}$$

By splitting the complex exponential into its real and imaginary parts and using the linear coordinate change from before, we actually obtain (cf. [6])

$$\begin{aligned} \sum _{\lambda \in \Lambda _2} \phi _{\alpha }(|\lambda |^2) e^{2 \pi i (J\lambda ) \cdot z_0}&= \sum _{\lambda \in \Lambda _2} \phi _{\alpha }(|\lambda |^2) \cos (2 \pi (J\lambda ) \cdot z_0)\\&= \sum _{(k,l)\in {\mathbb {Z}}^2} e^{- \frac{2 \pi \alpha }{\sqrt{3}} (k^2+kl+l^2)} \cos \left( 2 \pi (k-l)/3 \right) . \end{aligned}$$

Note that $\cos (2\pi (k-l)/3) \in \{-1/2, 1\}$ for all $(k,l) \in {\mathbb {Z}}^2$. Also, the way in which the values are taken is determined by the quadratic form $q(k,l) = k^2+kl+l^2$ and

$$\begin{aligned} \lim _{R \rightarrow \infty }\frac{|\{(k,l) \in {\mathbb {Z}}^2 \mid \cos (2\pi (k-l)/3) = -1/2\} \cap [-R,R]^2|}{|\{(k,l) \in {\mathbb {Z}}^2 \mid \cos (2\pi (k-l)/3) = 1\} \cap [-R,R]^2|} = 2. \end{aligned}$$

So we have twice as many negative signs with weight 1/2 in the series as positive signs (with weight 1), leading to an overall “neutral charge” of $\Lambda _2$ (cf. [7]). In fact, we can decompose $\Lambda _2$ into 3 relatively shifted copies (shifted by deep holes), 2 copies having negative signs with half weights, and 1 copy with positive signs (and full weights) (cf. [6]). This is the geometric interpretation (cf. [16]) of the *arithmetic part* of the cubic AGM of Borwein and Borwein [11]: for a complex number *q* with |q|<1, set

$$\begin{aligned} a(q) = \sum _{(k,l) \in {\mathbb {Z}}^2} q^{k^2+kl+l^2} \quad \text { and } \quad b(q) = \sum _{(k,l) \in {\mathbb {Z}}^2} q^{k^2+kl+l^2} e^{2 \pi i \frac{k-l}{3}}. \end{aligned}$$

Then

$$\begin{aligned} 3 a(q^3) = a(q) + 2b(q). \end{aligned}$$

Setting $q = e^{-\frac{2 \pi \alpha }{\sqrt{3}}} < 1$, α>0, we have

$$\begin{aligned} \sum _{\lambda \in \Lambda _2} \phi _{\alpha }(|\lambda |^2) e^{2 \pi i (J\lambda ) \cdot z_0} = b(q) = \frac{3 a(q^3)-a(q)}{2}, \end{aligned}$$

and

$$\begin{aligned} \sum _{\lambda \in \Lambda _2} \phi _{\alpha /2}(|\lambda |^2) e^{2 \pi (J\lambda ) \cdot z_0} = b(q^{1/2}) = \frac{3a(q^{3/2})-a(q^{1/2})}{2}. \end{aligned}$$

Similar to before, we make an auxiliary computation. We seek to find $x \in {\mathbb {R}}_+$ such that

$$\begin{aligned} 3 e^{-3x}- e^{-x} > 3e^{-\frac{3x}{2}} - e^{-\frac{x}{2}}. \end{aligned}$$

Substituting $s = e^{-x/2}$, this leads to finding s∈[0,1] such that

$$\begin{aligned} 3s^6-3s^3-s^2+s = s \ (3s^5-3s^2-s+1) = s \ g(s) >0. \end{aligned}$$

Clearly, the value of *g*(*s*) at s=0 is g(0)=1. Moreover, g(1/5)=2128/3125>0. We check the sign of the derivative. We have

$$\begin{aligned} g'(s) = 15 s^4 - 6s - 1. \end{aligned}$$

For 0<s<1/5, we have $$15s^4< 3/125 < 1$$ and so $g^{\prime }\left(s\right)<0$ on (0, 1/5). Thus *g*(*s*) is positive on the interval (0, 1/5) and, ultimately,

$$\begin{aligned} s \ g(s)> 0, \ s \in (0,1/5) \quad \Longleftrightarrow \quad 3 e^{-3x}- e^{-x} > 3e^{-\frac{3x}{2}} - e^{-\frac{x}{2}}, \ x \in (2 \log (5), \infty ). \end{aligned}$$

We note that our estimates are far from optimal, but they imply

$$\begin{aligned} \frac{3a(q^3)-a(q)}{2} {>} \frac{3a(q^{3/2})-a(q^{1/2})}{2}, \quad q {=} e^{-\frac{2 \pi \alpha }{\sqrt{3}}}, \quad \alpha {>} \frac{2 \log (5) \sqrt{3}}{2 \pi } \approx 0.88733 \end{aligned}$$

by comparing for all $(k,l) \in {\mathbb {Z}}^2 \setminus \{(0,0)\}$. Note that the value at the origin adds 1 to both sides of the inequality. Thus, the proof of Theorem 1.1 is finished. □
